# Endolog technique for correction of hallux valgus: a prospective study of 30 patients with 4-year follow-up

**DOI:** 10.1186/s13018-015-0245-1

**Published:** 2015-07-02

**Authors:** Carlo Biz, Marco Corradin, Ilaria Petretta, Roberto Aldegheri

**Affiliations:** Orthopaedic Clinic, Department of Surgery, Oncology and Gastroenterology DiSCOG, University of Padua, via Giustiniani 2, 35128 Padova, Italy

**Keywords:** Bunion, Hallux valgus, Endolog, Endomedullary nail, Distal osteotomy, First ray deformities

## Abstract

**Background:**

Hallux valgus (HV) is a complex deformity of the forefoot altering the kinematics of walking. Many different treatment alternatives exist for the correction of hallux valgus, but to date, none has been shown to be more effective than any other. The rate of complications following hallux valgus surgery is variable and has been reported as ranging from 1 to 55 % in the scientific literature. The purpose of this preliminary prospective study was to evaluate the result of the Endolog device, an innovative titanium endomedullary nail, for the treatment of HV.

**Methods:**

Thirty patients with mild-to-severe HV were treated with the Endolog device. Clinical evaluation was assessed preoperatively, as well as at 3, 6, 12, 24, and 48 months after surgery with a final follow-up at 4 years, using the American Orthopaedic Foot and Ankle Society (AOFAS) hallux grading system. Computer-assisted measurement of weight-bearing antero-posterior radiographs was taken preoperatively and postoperatively, as well as at 3, 6, 12, 24, and 48 months after surgery. Non-weight-bearing radiographs were taken before the patients were discharged. The radiological parameters measured included the intermetatarsal angle (IMA), the hallux valgus angle (HVA), the distal metatarsal articular angle (DMAA), and the tibial sesamoid position. Statistical analysis was carried out using the paired *t* test (*p* < 0.05).

**Results:**

The mean AOFAS score was 93.98 points at the 48-month follow-up. The postoperative radiographic assessments showed a statistically significant improvement compared with preoperative values. The mean corrections for each angular value at the last follow-up were as follows: IMA 5.95°; HVA 16.81°; DMAA 10.70°; and tibial sesamoid 1.36°.

**Conclusion:**

The Endolog is a safe and effective technique for the correction of HV deformity, to relieve pain and to preserve joint movement.

## Background

Hallux valgus (HV) is a complex deformity of the forefoot altering the kinematics of walking. In many cases, it is inherited and is found frequently in women between 40 and 60 years old living in western industrialized and developing countries [1, 2]. It is estimated to affect 23 % of adults and 35.7 % of elderly individuals [3]. Its pathogenesis is considered multifactorial, related both to constitutional and familial-hereditary factors, in particular to an imbalance in the abductor and adductor muscles, as well as to the use of constrictive footwear, particularly tight-fitting, high-heeled shoes [4].

HV is considered a progressive deformity characterized by subluxation of the first metatarsophalangeal joint (MTPJ) with lateral displacement and pronation of the big toe, metatarsus varus, exostosis of the metatarsal head, and instability of the first tarsometatarsal joint (TMTJ). It is often associated with callus, bursa over the bony prominence, and lesser toe deformities [5]. A wide variety of surgical procedures have been described in the literature [6] for its correction including first metatarsal osteotomies, osteotomy of the first cuneiform, Lapidus arthrodesis, and fusions [7, 8]. Because none has been shown to be more effective than any other, more than 200 different surgeries have been designed [9]. An innovative technique using the “Endolog” device has recently been proposed for mild to moderate and even for severe forms.

The purpose of this prospective study was to evaluate the validity and reliability of the Endolog technique for correction of mild-to-severe HV after a 4 year follow-up with regards to functional results, first MTP joint stiffness, and patient satisfaction, as well as clinical and radiographical evidence of correction of the deformity.

## Material and methods

### Patients

All subjects participating in this study received a thorough explanation of the risks and benefits of inclusion and gave their oral and written informed consent to publish the data. Approval from the General Clinic Directorate of our institution was obtained before using the Endolog device and starting the analysis. The study was performed in accordance with the ethical standards of the 1964 Declaration of Helsinki as revised in 2000. Between May 2008 and May 2009, 30 feet of 30 consecutive patients (28 females and 2 males) underwent the Endolog procedure for treatment of hallux valgus at our Orthopaedic and Traumatology Clinic. All 30 operative procedures were performed by a single surgeon, the senior author (CB).

### Inclusion and exclusion criteria

The patients with diagnosis of mild-to-severe HV were enrolled consecutively with precise inclusion criteria over a 1 year period. All patients considered in this study had to be between 35 and 75 years of age, suffering from mild-to-severe HV deformity and complaining of constant pain in the area of the first metatarsal head or isolated to the first MTP joint region, and having particular discomfort when wearing shoes. In fact, pain was the primary indication for the surgical treatment, and not one patient was operated on for cosmetic reasons. Specific patient exclusion criteria were as follows: history of previous foot surgery or trauma, diagnosis of diabetes mellitus, rheumatological diseases or psoriatic arthritis, foot neuropathy, vascular insufficiency, generalized joint laxity or hypermobility of the first ray more than 10 mm, and hallux rigidus. Furthermore, patients were excluded if they had interphalangeal hallux valgus, fixed lesser toe deformities, or associated deformities in the joint of the foot. Finally, none was receiving specific pharmacological treatment before the operation, such as the most common non-steroidal anti-inflammatory medications or injections.

### The Endolog device and surgical techniques

**Fig. 1:**
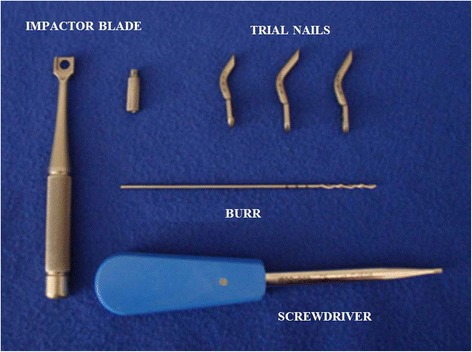
The complete kit of the Endolog device

The Endolog, produced since 2006 by Medical2, Castelnuovo del Garda, Verona, Italy, is a curved titanium endomedullary nail device (TA6V ELI - ASTMF 136), treated with anodic oxidation and laser marking. It is formed by a curvilinear cylindrical body with a diameter of 4.5 mm and a blade inclined by 4° with respect to the axis of the nail, which serves to push for lateral translation of the metatarsal head. The Endolog is available in three sizes (44, 45, and 46) with three different degrees of curvature (32°, 40°, and 42°) and three different lengths (26, 31, and 33 mm). It is fixed to the metatarsal head using a 3.66-mm titanium angular stable screw, available in three different lengths (15, 20, and 25 mm), which stabilizes the osteotomy sides and the translation of the metatarsal head (Fig. 1). The term Endolog was coined by its inventor Giuseppe Lodola with reference to the endomedullary component of the nail (*Endo*) and his own initials (*Lo-G*). It is provided with dedicated instruments that include an impactor with a special drill guide, three test sizes of the nail, a graduated drill tip, and a screwdriver. These features give the device maximum biocompatibility, no interference in case of MR scan investigation, absolute sterility, traceability of the system, and adherence to the European legal regulations (93/42CE). As far as we are aware, no other available device has the unique technical characteristics of this nail, apart from the predecessor of the Endolog, the “hallux splint” [10].

Prophylactic antibiotic (cefazolin 2 g) was administered before surgery, and thromboembolic prophylaxis with nadroparin calcium was prescribed the same evening for a 10 day period. Anesthesia consisted in a regional foot block, which combines five nerves, three superficial (saphenous, sural, and superficial peroneal nerves) and two deep (tibial and deep peroneal nerves). A tourniquet was applied and left in place at the level of the ankle. A 4-cm dorsal-medial longitudinal incision was made at a point corresponding to the exostosis of the first metatarsal, avoiding the dorsal digital branch of the medial cutaneous nerve, and the neurovascular bundle was protected appropriately. Then, the capsular incision was performed in a dorsal longitudinal orientation along the line of the skin incision. Capsular and ligamentous tissues were freed around the first metatarsal head dorsally and medially, and the bone was liberated from the periosteum.

**Fig. 2:**
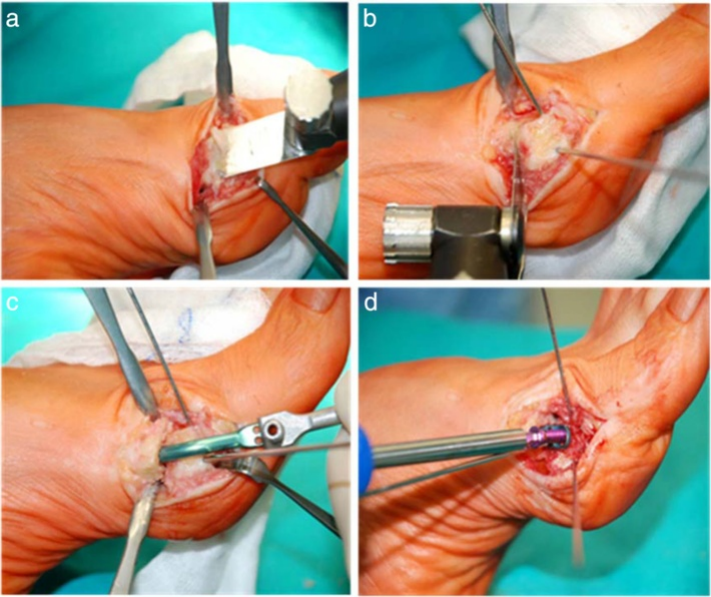
**a** Exostosectomy. **b** Lateral translation of the metatarsal head. **c** Application of the Endolog device. **d** Endolog fixed with screw

**Fig. 3:**
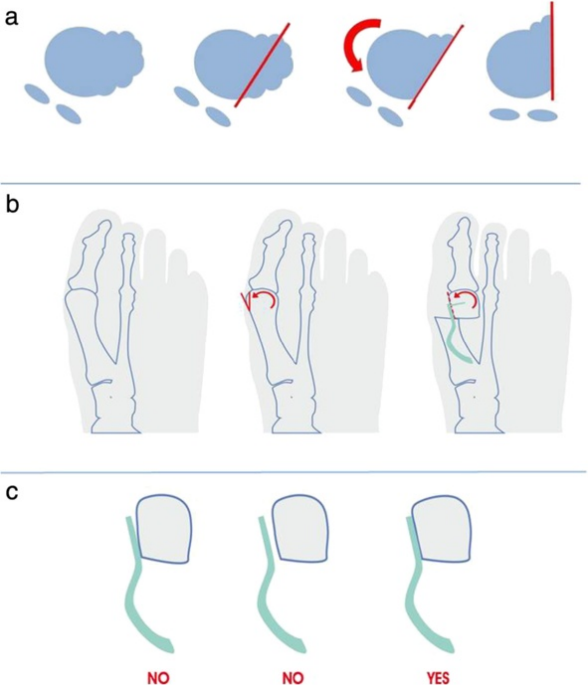
**a**–**b** The oblique exostosectomy will permit the correction of the DMAA value and sesamoid subluxation during the subsequent derotation and translation of the metatarsal head while the nail is inserted after osteotomy at the neck level. **c** Perfect coplanarity and maximum adherence of the pallet support to the flat surface of the metatarsal head

Using a standard oscillating saw in a distal to proximal direction, a very minimal, oblique exostosectomy was performed to remove the medial eminence and to produce a flat surface on the head in order to support the impactor’s blade upon which the device was assembled (Figs. 2a and 3a, b). For a correct position of the device, perfect coplanarity and maximum adherence of the pallet support to the flat surface previously created on the metatarsal head is crucial (Fig. 3c). The oblique exostosectomy was carried out with a thickness of no more than 2–4 mm from the distal part of the medial eminence, close to the articular surface, to zero at the level of the metatarsal neck, making a lateral translation of the head possible, pushed and maintained by the nail after its application, and correcting both the DMAA and the dislocated sesamoid apparatus due to pronation of the big toe during the following derotation of the metatarsal head (Fig 3a, b). For this purpose, two 1.8-mm Kirschner wires, acting as joysticks, were inserted to allow the derotation of the metatarsal head during its lateral translation. A linear osteotomy, at times perpendicular to the proximal level of the neck and at times oblique in order to lengthen or to shorten the metatarsal, was performed (Fig. 2b). Once the trial Endolog device was assembled on the impactor, it was gently introduced into the medullary cavity with progressively lateral displacement of the head and contemporary derotation of the metatarsal head, using the K-wires like joysticks and correcting the DMAA and sesamoid subluxation (Fig. 2c).

The correction attained was checked clinically and under fluoroscopy before the final device was applied. The correction and the implant were stabilized applying temporary 1.2-mm Kirschner wires through the holes of the device. The head was fixed to the implant with a screw long enough to provide angular stability (Fig. 2d). Once the wire was removed and before closing the capsule and suturing the skin with 2–0 reabsorbable stitches, it was necessary to regulate the medial angle of the metatarsal neck in order to prevent conflict of the bone with the soft tissues and skin. A compression dressing and tape were applied to maintain a slight hypercorrection of the hallux; these were changed weekly. Finally, the duration of the surgery was recorded. In order to study only the efficacy of the Endolog technique on correction of HV deformity, no soft-tissue procedures, such as adductor hallucis tendon release or lateral capsulotomy, were performed.

### Postoperative protocol

All patients had the same postoperative regime and were followed in the same standardized manner by the senior author (CB). Patients were seen within 12 h, and the gauzes and tape compression dressing were changed. The patients were allowed to walk as tolerated the day after surgery using a heel-bearing shoe for the following 30 day period. Antero-posterior and lateral X-rays of non-weight-bearing feet were taken before the patients were discharged and at 1 month follow-up, but they were not included for the radiographic evaluation, the first because they were non-weight-bearing and the latter because of the presence of the bandaging. All of the patients were seen once a week for a month in our outpatient clinic where the functional taping was replaced at the first three appointments.

### Clinical and radiological evaluation

**Fig. 4:**
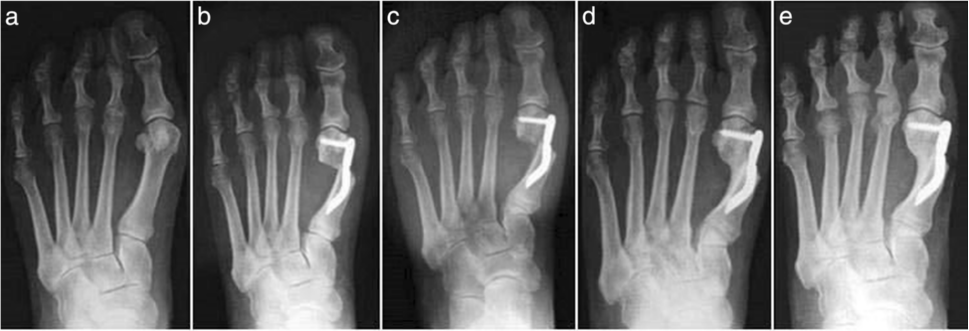
Case 1. A 44-year-old woman with mild hallux valgus (HV). **a** Preoperative antero-posterior radiographic image. **b** Endolog translation more than 100 % at 1-month follow-up, X-ray image. **c** Bone callus formation at 3-month follow-up, X-ray image. **d** a at 6-month follow-up X-ray image. **e** Radiographic aspect at 48-month follow-up showing the maintained correction of the deformity and complete healing of the osteotomy

The clinical and radiological analyses were carried out by two independent investigators, the junior authors (MC and IP) not involved in the treatment of the patients. The preoperative evaluation included a complete clinical history of the patients, physical examination of the foot, and routine standing antero-posterior, lateral X-ray and sesamoid X-ray views. For this study, all of the patients underwent clinical and radiographic assessment before surgery, as well as at 3, 6, 12, and 24 months after surgery and at the final follow-up of 48 months (Fig. 4), using the following:

- The hallux-metatarsophalangeal scale, proposed by the American Orthopaedic Foot and Ankle Society (AOFAS) [11].
- The Med Station program (X-ray data base of our hospital). This software allows the retrieval of electronically computer-assisted measurements from weight-bearing radiographs [12–14] (non-weight-bearing radiographs were taken before the patients were discharged) of the following angles: the intermetatarsal angle (IMA) (normal value <10°), the distal metatarsal articular angle (DMAA) (normal value <6°), and the metatarsophalangeal (hallux valgus angle (HVA)) angle (normal value <15°).
- The classification system recommended by the AOFAS [15, 16] evaluating the tibial sesamoid position.

The relationship between the IMA, HVA values, and tibial sesamoid displacement was used to classify the deformities into three groups according to the presence of one of these Mann and Coughlin parameters [15–19]:

- Mild HV was defined as an IMA ≤11° and HVA <20° and less than 50 % subluxation of the medial sesamoid (grade 1).
- Moderate HV was an IMA >11° but <16° and HVA of 20° to 40°, with 50 to 75 % subluxation of tibial sesamoid (grade 2).
- Severe HV was an IMA ≥16° and HVA of >40° and more than 75 % subluxation of tibial sesamoid (grade 3).

### Statistical analysis

The IMA, DMAA, and HVA values were analyzed with mixed models for repeated measurements considering an unstructured variance-covariance matrix. The least squares mean and the 95 % confidence interval were estimated at each time point. The statistical level of significance was set at 5 %.

## Results

**Fig. 5:**
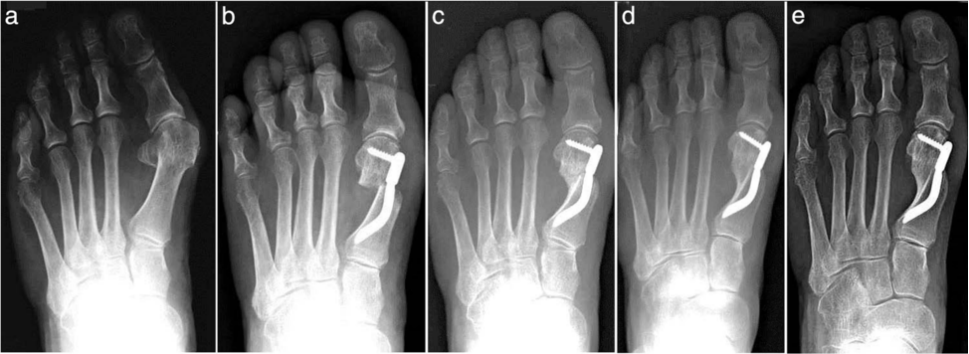
Case 2. A 64-year-old woman with moderate HV; antero-posterior radiographic images at: **a** Preoperative period. **b** 1-month follow-up. **c** 3-month follow-up. **d** 6-month follow-up. **e** 48-month follow-up showing the maintained correction of the deformity

**Fig. 6:**
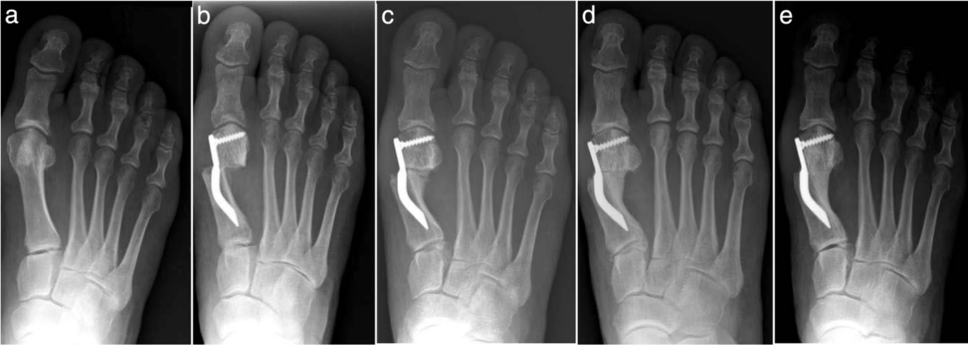
Case 3. A 57-year-old woman with mild HV; antero-posterior radiographic images at: **a** Preoperative period. **b** 1-month follow-up. **c** 3-month follow-up. **d** 6-month follow-up. **e** 48-month follow-up showing the maintained correction of the deformity

**Fig. 7:**
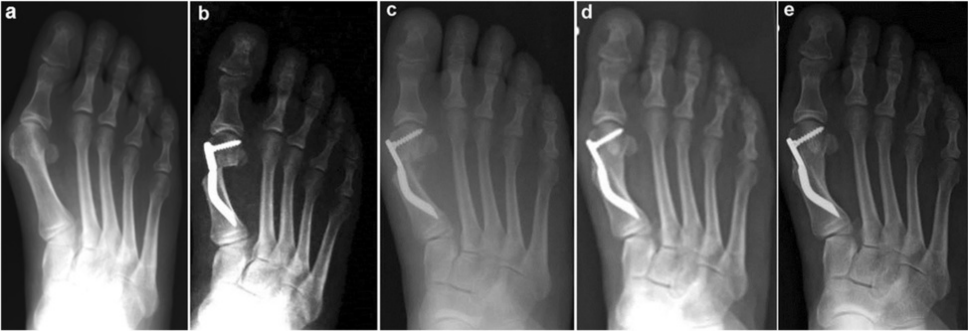
Case 4. A 51-year-old woman with moderate HV; antero-posterior radiographic images at: **a** Preoperative period. **b** 1-month follow-up. **c** 3-month follow-up. **d** 6-month follow-up. **e** 48-month follow-up showing the maintained correction of the deformity

**Fig. 8:**
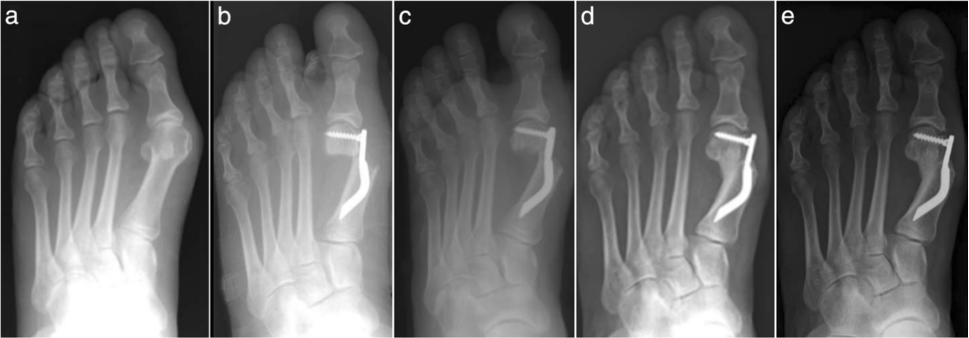
Case 5. A 52-year-old woman with moderate HV; antero-posterior radiographic images at: **a** Preoperative period. **b** 1-month follow-up. **c** 3-month follow-up. **d** 6-month follow-up. **e** 48-month follow-up showing the maintained correction of the deformity

**Fig. 9:**
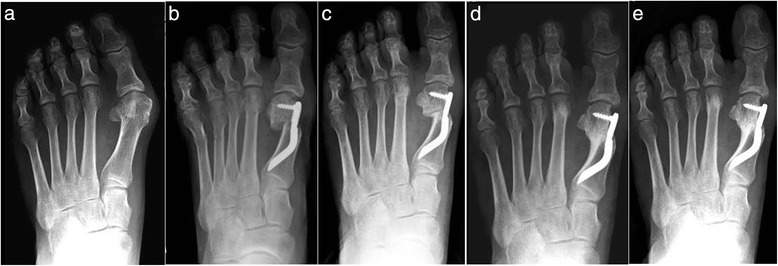
Case 6. A 54-year-old woman with moderate HV; antero-posterior radiographic images at: **a** Preoperative period. **b** 1-month follow-up. **c** 3-month follow-up. **d** 6-month follow-up. **e** 48-month follow-up showing the maintained correction of the deformity

**Fig. 10:**
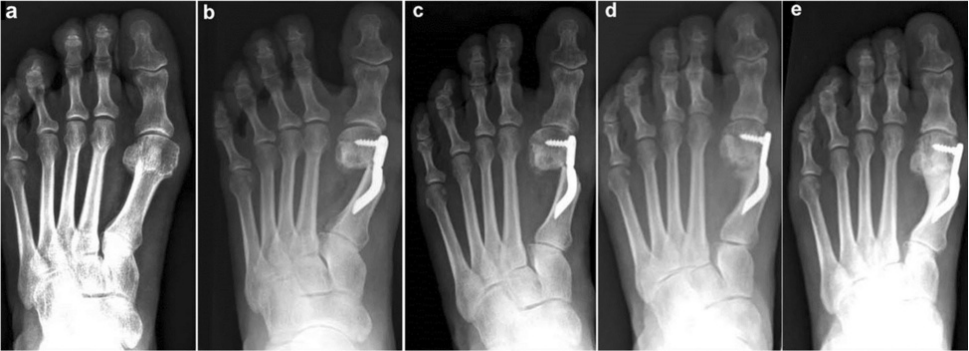
Case 7. A 55-year-old woman with mild HV; antero-posterior radiographic images at: **a** Preoperative period. **b** 1-month follow-up. **c** 3-month follow-up. **d** 6-month follow-up. **e** 48-month follow-up showing the maintained correction of the deformity

**Fig. 11:**
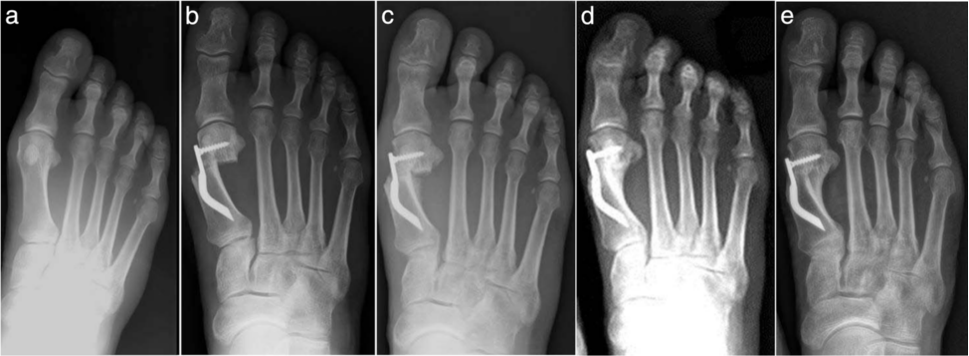
Case 8. A 38-year-old woman with mild HV; antero-posterior radiographic images at: **a** Preoperative period. **b** 1-month follow-up. **c** 3-month follow-up. **d** 6-month follow-up. **e** 48-month follow-up showing the maintained correction of the deformity

**Fig. 12:**
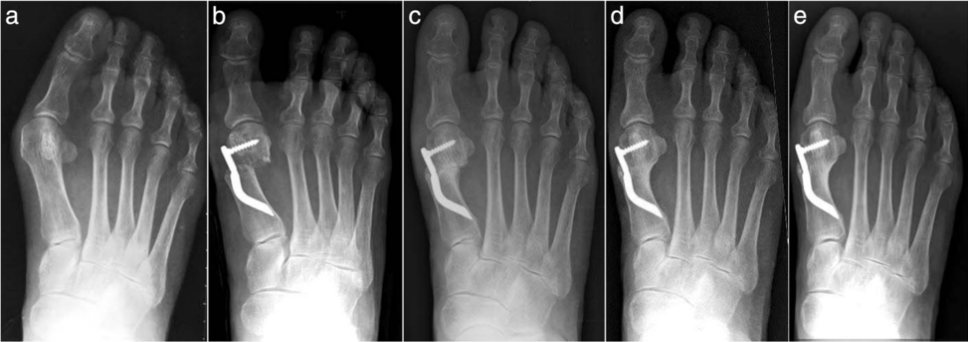
Case 9. A 71-year-old woman with moderate HV; antero-posterior radiographic images at: **a** Preoperative period. **b** 1-month follow-up. **c** 3-month follow-up. **d** 6-month follow-up. **e** 48-month follow-up showing the maintained correction of the deformity

**Fig. 13:**
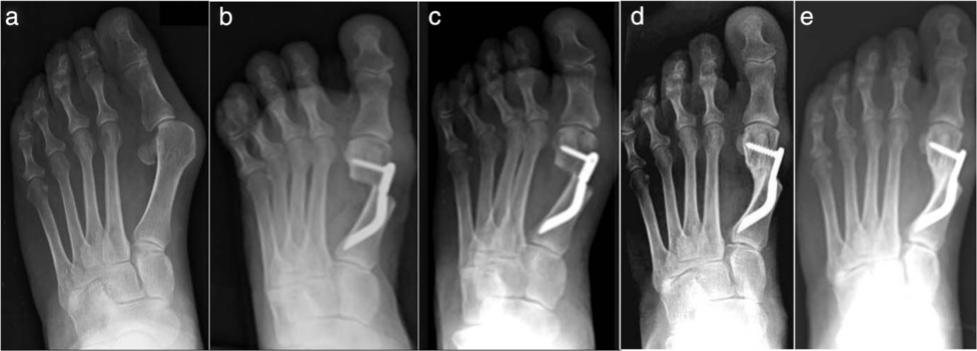
Case 10. A 59-year-old woman with severe HV; antero-posterior radiographic images at: **a** Preoperative period. **b** 1-month follow-up. **c** 3-month follow-up. **d** 6-month follow-up. **e** 48-month follow-up showing the maintained correction of the deformity

Thirty feet, 16 rights and 14 lefts, of 30 consecutively enrolled patients, met the inclusion criteria and were considered in the analyses. The median patient age at the time of the surgery was 56.5 (range 38 to 73 years). There were 28 women (93.3 %) and 2 men (6.7 %). None of the patients was lost during the different follow-ups including the final one. According to the Mann and Coughlin grading system, 11 (36.7 %) patients fell into group A (mild), 14 (46.6 %) into group B (moderate), and 5 (16.7 %) into group C (severe). In Figs. 5, 6, 7, 8, 9, 10, 11, 12, and 13, antero-posterior radiographic images of a series of nine cases are presented. For each case, the following are shown: (a) the preoperative radiographic aspect of the deformity, as well as the postoperative radiographic images showing the first metatarsal head translation, the maintained correction of the deformity, and the progressive formation of the bone callus until the complete healing of the osteotomy at the different follow-ups of (b) 1, (c) 3, (d) 6, and (e) 48 months (Figs. 5, 6, 7, 8, 9, 10, 11, 12, and 13).

**Fig. 14:**
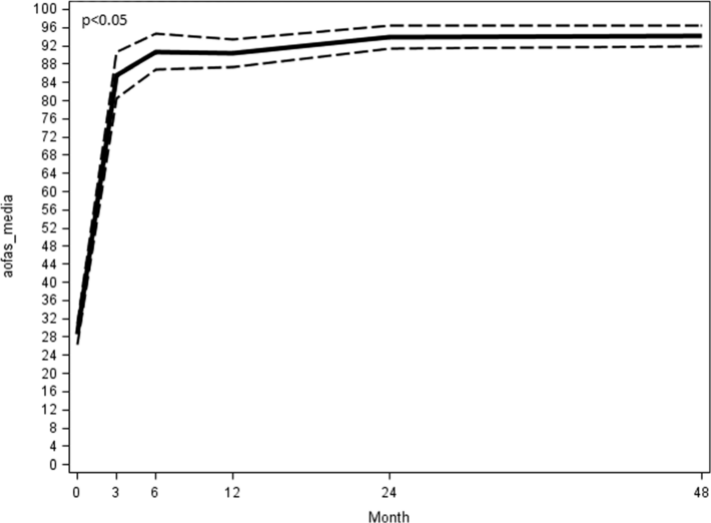
AOFAS preoperative score at different follow-ups

The mean total AOFAS score of the patients treated was 28.7/100 points (range 19–42) at the preoperative evaluation, and it significantly and progressively improved (*p* <0.05) at the different follow-ups until the final available: 85.53 points (range 44–100) at 3-month follow-up; 90.67 at 6-month follow-up; 90.47 at 12 month follow-up; 93.93 at 24 month follow-up; and 93.98 at the final 48 month follow-up (Fig. 14).

The symptomatic hyperkeratosis under the first metatarsal head found in 19 patients (63.3 %) at the preoperative evaluation was completely resolved at follow-up. The average surgical time of procedure, from skin incision to taping application, was 39 min (range 31–46).

### The intermetatarsal angle (IMA)

**Fig. 15:**
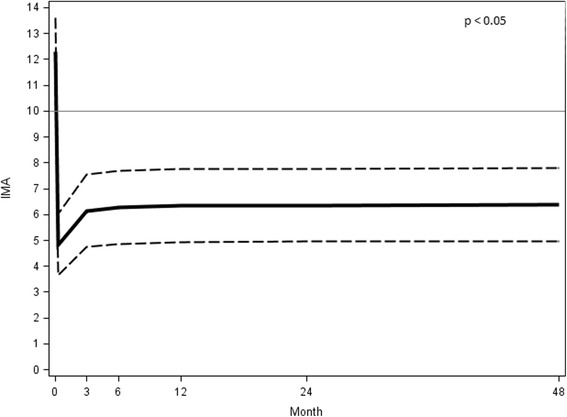
IMA preoperative angular value at different follow-ups

The mean IMA value decreased from 12.31° ± 3.05° (range 7.09°–18.55°) preoperatively to 6.36° ± 1.38° (range 7.74°–4.98°) at the 48 month follow-up with a mean correction of 5.95° (*p* < 0.05) (Fig. 15).

### The hallux valgus angle (HVA)

**Fig. 16:**
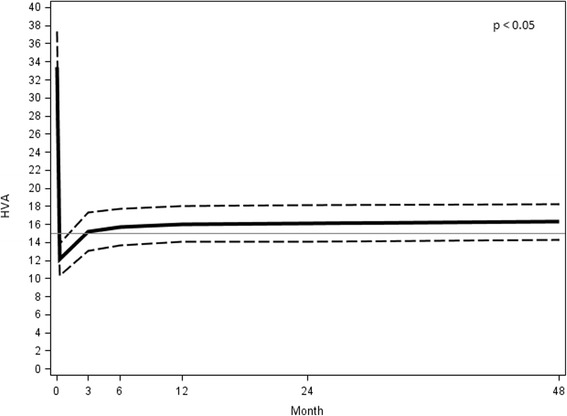
HVA preoperative angular value at different follow-ups

The mean preoperative HVA was 33.38° ± 10.72° (range 17.84°–66.62°). The mean value at the 48 month follow-up assessment was 16.57° ± 5.40° (range 7.18°–25.18°) with a mean correction of 16.81° (*p* < 0.05) (Fig. 16).

### The distal metatarsal articular angle (DMAA)

**Fig. 17:**
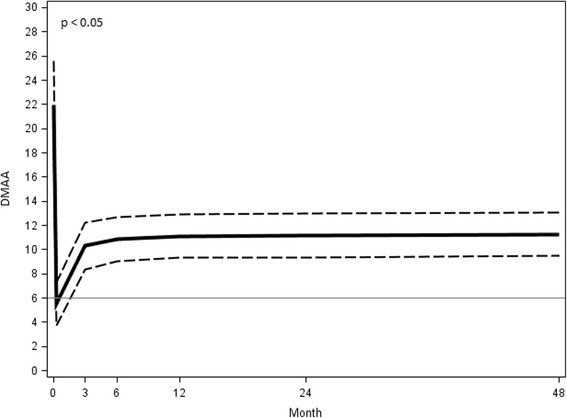
DMAA preoperative angular value at different follow-ups

The mean preoperative DMAA was 21.95° ± 9.77° (range 8.09°–43.22°). The mean value at the 48 month follow-up examination was 11.25° ± 4.79° (range 3.84°–22.10°) with a mean correction of 10.70° (*p* < 0.05) (Fig. 17).

### The medial sesamoid position

The mean preoperative dislocation of the medial sesamoid was 2.83° (range 2°–3°). Its mean value at the 48 month follow-up assessment was 1.47° (range 0°–3°) with a mean correction of 1.36° (*p* < 0.05).

**Fig. 18:**
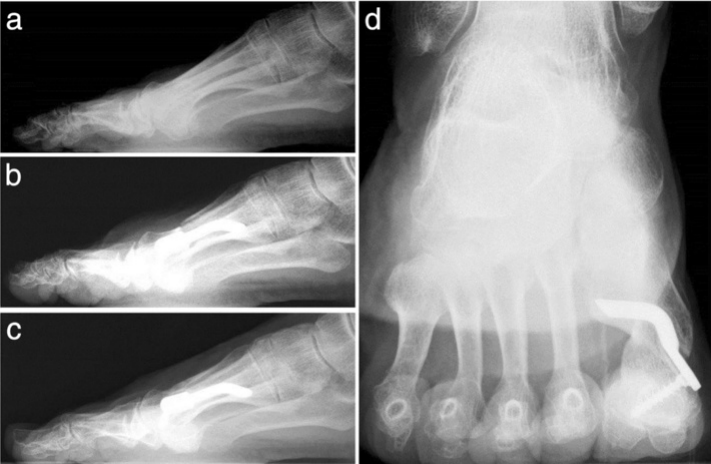
Case 12. A 71-year-old woman with moderate HV. **a** Preoperative lateral radiographic image. **b** At 1-month follow-up, lateral X-ray image. **c** Radiographic aspect at 48-month follow-up showing the maintained correction of the deformity and complete healing of the osteotomy. **d** X-ray image, sesamoid axial view, at 48-month follow-up

Regarding complications, there were no cases of superficial wound infection, cellulitis, osteomyelitis, or postoperative lesser transfer metatarsalgia. None of the 30 patients experienced postoperative avascular necrosis of the first metatarsal head. We did not encounter any recurrence of deformity, hallux varus due to overcorrection, malunion, delayed union, non-union, residual pain, or irritation from internal fixation by the device (Fig. 18). None of the first 30 implants was removed after the 4 year follow-up.

## Discussion

The treatment of hallux valgus is still debated; more than 160 surgical techniques have been described, and the choice depends on the surgeon’s experience [19–21]. Commonly, distal first metatarsal osteotomies (Austin, Chevron, Reverdin’s variant, or Mitchell) are used for treatment of mild deformities, while severe ones are best treated by proximal osteotomies in addition to soft-tissue release [19]. In any case, radiographs are always essential in the preoperative assessment and in the choice of the most appropriate procedure [20]. Multiple retrospective studies have reported the results of many different techniques proposed for HV correction, but, to the best of our knowledge, this is the first prospective study reporting the outcome of the Endolog technique at the 4 year follow-up. Only one retrospective study [22] showing midterm outcome of this device has been published.

This prospective study was designed to evaluate, on the basis of clinical and computer-assisted radiographic [12] data of a series of 30 patients prospectively followed up for 4 years, the validity and reliability of the Endolog technique for correction of moderate-to-severe HV deformity.

In our patient group, the result after a 4 year follow-up showed a significant improvement in the AOFAS score and radiographic parameters than the preoperative score. In accordance with other investigators’ reports [10, 16, 20–23], it has been found that an internal device such as the Endolog, which ensures a stable, but not rigid synthesis of distal osteotomy, is linked to a significant improvement in all of the clinical parameters assessed and in particular in pain alleviation, alignment, and elimination of plantar keratotic lesions. In fact, with a median AOFAS score of 93.98 points at the 4 year follow-up, the clinical results obtained with the Endolog technique for the correction of mild-to-severe HV deformity are comparable to those obtained with other common procedures such as Chevron, Scarf, or proximal metatarsal osteotomies [24–26], and Lapidus bunionectomy [27]. One of the possible side effects of hallux valgus surgery is rigidity of the first metatarsophalangeal joint [28, 29], but it has been found that the technical characteristics of the Endolog nail, completely endomedullary except for a 5-mm blade disposed on the head, result in a transitory minimal stiffness. After 3 months, 13 (43.3 %) of the patients had recuperated more than 75 % articulation, and only one presented severe limitation (<30 %). Hence, the postoperative stiffness with this procedure is comparable or inferior to the stiffness reported for other open [24, 29, 30] or percutaneous techniques [28].

Alignment was considered good in 21 cases (70 %), discrete in 7 (23.4 %) cases, poor in 2 (6.6 %) cases, and without significant variations at the follow-up. This is an encouraging finding, especially considering the severity of the cases treated; it could be explained by the stability ensured by the implant. No cases of shortening of the first metatarsal, malposition, recurrence, delayed or non-union, transfer metatarsalgia, deep infection of soft tissues, osteomyelitis, or avascular necrosis of the metatarsal head (which is one of the most serious complications of HV surgical correction by distal osteotomy, along with infection) were recorded in the patients studied [31–33]. As the fixation is completely internal, the risk of infection is greatly reduced with respect to other techniques [24, 28] that use percutaneous K-wires. There were no sequelae associated with metallic fixation, such as painful tissue irritation, loosening, or breakage of the implant. In fact, none of this first series of thirty Endologs has been removed to date.

The choice of procedure depends principally on the severity of the deformity. For correction of mild-to-moderate HV, several distal osteotomies have been reported to yield good clinical results, including the Chevron [34], Austin, Wilson, and Mitchell techniques. However, only a relative small amount of correction of the deformity is possible, and some shortening of the first metatarsal results with consequent risk of avascular necrosis of the metatarsal head [35] and transfer metatarsalgia. Instead, proximal osteotomies, which are usually also performed with a lateral soft-tissue release, are recommended for the treatment of moderate to severe deformities because they allow a greater degree of correction [34, 35]. These include proximal Chevron, Ludloff osteotomy, crescentic osteotomy as described by Mann [2], and Scarf osteotomy. In particular, Scarf osteotomy has become popular because of its inherent stability, minimal shortening of the first metatarsal, and ease of internal fixation [34]. Some authors believe that the use of combined techniques during the same procedure gives better results. However, our results support the effectiveness of only one procedure for different degrees of HV severity. The main expectations of the patients before HV surgery are pain relief and improvement in footwear and walking ability [36–39]. Our clinical and radiographic results following surgery and at the follow-ups satisfied the patients’ expectations. In fact, the multiplanarity of the correction (IMA), the derotation of the metatarsal head (DMAA), the clinical correction of valgism (HVA), and the anatomic reduction of the tibial sesamoid, necessary to prevent the recurrence of valgism [40], were all maintained. In particular, the DMAA correction is due to triplanar movement of the metatarsal head after osteotomy exerted by the device during its gradual application and is maintained by the screw fixation in the desired position with the Endolog system.

From a surgical viewpoint, using the Endolog device allows a direct view of the metatarsal head so that its translation and derotation can be controlled on the frontal and sagittal planes. The main advantages over other surgical techniques are a simpler procedure, lower learning curve, shorter surgery time (less than 40 min), reduced risk of complications, smaller incisions, shorter hospital stays, and immediate recovery of walking.

The strength of our study is its prospective evaluation in a group of patients with the same fixed follow-ups until 4 years. The operations and aftercare were performed in the same way by a single surgeon (CB). This series represents his second series of 30 implants. All patients were examined clinically and radiographically by two independent investigators (MC and IP), not directly involved in the patients’ treatment, during the follow-up of 4 years. The absence of associated lesser toe deformities in the treated selected feet has allowed us to test only the efficacy of the Endolog in correcting HV without conditioning the final results and reducing the bias. We are aware of the limitations of the present study. The main one is the small group of the patients, although the number of 30 feet is common in the foot and ankle surgery papers in the literature. Further, we lacked a control group, with which it would be useful to compare the results of our method. Hence, we believe that long-term follow-ups with larger cohorts and randomized controlled clinical trials comparing the Endolog technique to other methods are necessary to provide useful information for foot and ankle surgeons.

## Conclusions

Although it is accepted that a single surgical procedure is not able to cover the wide range of deformities of hallux valgus, the Endolog technique is a feasible procedure that extends the surgical indications to even more severe forms. The device allows a wide variability of correction, according to its three different sizes. Finally, our data suggest that, in the short- and medium-term, the Endolog technique appears to be a safe, effective, and reliable procedure for correction of moderate-to-severe HV deformity with a low occurrence of complications and no recurrence.
